# Clinical and biometrical 12-month follow-up in patients after reconstruction of the sural nerve biopsy defect by the collagen-based nerve guide Neuromaix

**DOI:** 10.1186/s40001-017-0279-4

**Published:** 2017-09-22

**Authors:** Ahmet Bozkurt, Kristl G. Claeys, Simone Schrading, Jana V. Rödler, Haktan Altinova, Jörg B. Schulz, Joachim Weis, Norbert Pallua, Sabien G. A. van Neerven

**Affiliations:** 1 Department of Plastic Surgery, Reconstructive and Hand Surgery, Burn Center, RWTH Aachen University Hospital, Aachen, Germany; 2 Department of Plastic & Aesthetic, Reconstructive & Hand Surgery, Center for Reconstructive Microsurgery and Peripheral Nerve Surgery (ZEMPEN), Agaplesion Markus Hospital, Johann Wolfgang von Goethe University, Frankfurt, Germany; 3 Department of Neurology, RWTH Aachen University Hospital, Aachen, Germany; 4 Institute of Neuropathology, RWTH Aachen University Hospital, Aachen, Germany; 5 Department of Neurology, University Hospitals Leuven and University of Leuven (KU Leuven), Louvain, Belgium; 6 Department of Diagnostic and Interventional Radiology, RWTH Aachen University Hospital, Aachen, Germany; 7 Department of Neurosurgery, RWTH Aachen University Hospital, Aachen, Germany; 8 Jülich-Aachen Research Alliance (JARA)-Translational Brain Medicine, Forschungszentrum Jülich GmbH and RWTH Aachen University, Aachen, Germany; 9 Jülich-Aachen Research Alliance (JARA)-Institute Molecular Neuroscience and Neuroimaging, Forschungszentrum Jülich GmbH and RWTH Aachen University, Aachen, Germany; 10 Institute of Neuroscience, Université Catholique de Louvain, Avenue Mounier 53, 1200 Brussels, Belgium

## Abstract

Many new strategies for the reconstruction of peripheral nerve injuries have been explored for their effectiveness in supporting nerve regeneration. However only a few of these materials were actually clinically evaluated and approved for human use. This open, mono-center, non-randomized clinical study summarizes the 12-month follow-up of patients receiving reconstruction of the sural nerve biopsy defect by the collagen-based nerve guide Neuromaix. Neuromaix was implanted as a micro-structured, two-component scaffold bridging 20–40 mm nerve defects after sural nerve biopsy in twenty patients (eighteen evaluated, two lost in follow-up). Safety of the material was evaluated by clinical examination of wound healing. Performance was assessed by sensory testing of modalities, pain assessment, and palpation for the Hoffmann–Tinel’s sign as well as demarcating the asensitive area at each follow-up visit. Every patient demonstrated uneventful wound healing during the complete 12-month time course of the study. Two patients reported complete return of sensation, whereas eleven out of eighteen patients reported a positive Hoffmann–Tinel’s sign at the lower leg with simultaneous reduction of the asensitive area by 12 months. Our data show that Neuromaix can be implanted safely in humans to bridge sural nerve gaps. No procedure-related, adverse events, or severe adverse events were reported. These first clinical data on Neuromaix provide promising perspectives for the bridging of larger nerve gaps in combined nerves, which should be investigated more through extensive, multi-center clinical trials in the near future.

**Electronic supplementary material:**

The online version of this article (doi:10.1186/s40001-017-0279-4) contains supplementary material, which is available to authorized users.

## Background

Peripheral nerve injury (PNI) can have devastating consequences for patients when reconstructive strategies prove unsatisfactory [1]. Patients not only suffer from the functional motor, autonomic, or sensory loss caused by the disconnection of the nerve, they often develop secondary complications of which chronic neuropathic pain is the most disabling [2, 3].

In clinical practice the autologous nerve transplantation (ANT) is still regarded as the clinical gold standard to repair complex and extensive nerve injuries [4]. Yet the amount of autologous nerve material suitable for transplantation is limited in the human body. To reconstruct, most commonly the sensory sural nerve (SN) is harvested and implanted to bridge the nerve gap caused by the trauma [4]. Moreover harvesting the SN unquestionably leads to functional loss at the donor site, frequently followed by secondary complications, such as wound healing problems and chronic neuropathic pain [3]. Frequently, the autologous nerve material harvested is not sufficient to cover the complete diameter of the damaged nerve. Thus even after reconstruction by ANT, functional recovery is certainly not guaranteed in each case [1].

It is therefore not surprising, that in the last decades many new strategies and a large number of alternatives for the reconstruction of PNI have been explored for their effectiveness in supporting nerve regeneration. For example, other autologous materials [5–7] (e.g., veins-, muscle-, fat-grafts, and processed nerves from human corpses), non-degradable [8] (e.g., silicon, or poly-glycolic acid), and biodegradable or bio-derived materials (e.g., collagen, poly-lactide, chitosan) with micro-guidance structures have been tested pre-clinically for their merit in supporting nerve regeneration, most commonly in rodent models of PNI [9–18]. However only a few of these materials were actually clinically evaluated and approved for human use [19, 20]. From design perspective, these materials exist as hollow nerve tubes connecting both nerve stumps by an empty tube lumen. These materials were most often used for bridging small defects in sensory nerves (i.e., most commonly in the hand), where they proved to be effective in leading to some degree of sensitivity. However the bridging of larger nerve defects has remained a challenge. It was suggested that for effective bridging, additional microstructures within the tube lumen were required [21].

Currently, new strategies aim at developing such microstructures using various scaffold designs. For example, it was claimed that human donor nerve allografts retain their connective tissue microstructures and are already clinically used to bridge nerve gaps varying of 5–50 mm in sensory, motor, and combined nerves [22, 23]. Twenty-nine out of 42 acute repairs were performed in sensory nerves. From these acute repairs, the response rate, which was defined as any improvement in quantitative and/or qualitative data investigated, was 92.9%. Moreover similar response rates for the repair of sensory branches of the facial nerve have been reported [24]. So far this allograft is the only clinically used scaffold to our knowledge, providing microstructures to regenerate nerves.

The difficulties of translating animal data into the clinical setting have been emphasized lately [25]. Therefore, we introduced the medial SN biopsy model to investigate newly developed nerve guides in human PNI [20, 25]. In the standard procedure, the nerve gap that arises from nerve biopsy cannot be reconnected without tension. Reconstruction by ANT would be pointless in this case, as sensory loss at the donor site would be the trade-off [33]. When such an overcritical nerve gap persists, this often results in a permanent sensory loss at the lateral aspect of the foot. SN biopsies thus provide a very standardized human nerve injury model to test the biocompatibility and regeneration supporting potential of newly developed nerve guides [25]. Therefore in this current study we implanted the collagen-based, micro-structured nerve guide “Neuromaix” into 20–40 mm nerve gaps caused by medial SN biopsy in patients. Patients were followed until 12 months after surgery. Safety as well as performance parameters (i.e., sensory testing of different sensory modalities) were quantified for every single follow-up visit.

## Methods

### Screening and patient selection

After obtaining the patients’ written informed consent, a total number of 20 patients, in whom a diagnostic nerve biopsy has been performed, were immediately treated with the nerve guide Neuromaix. These patients, with a peripheral neuropathy of unclear origin, were selected after detailed neurologic history and physical examination, nerve conduction studies, and appropriate diagnostic work-up for peripheral neuropathy at the Neuromuscular Clinic of the Department of Neurology at the RWTH-Aachen University Hospital. Inclusion and exclusion criteria for this study are summarized in Table 1.

**Table 1:** In- and exclusion criteria

Inclusion criteria	Exclusion criteria
Patients between 18 and ≤ 70 years	Paraneoplastic pnp Alcohol-related polyneuropathy (pnp) Current immunosuppressive therapy Malignant tumor Peripheral vascular disease Collagenous diseases Diabetes mellitus Chronic venous insufficiency Deep vein thrombosis Skin diseases in the lower extremity Coagulopathy or anticoagulant therapy Pregnancy Infectious diseases (hiv, hepatitis)
Patients with clinical and electrophysiological suspect of a peripheral neuropathy that were indicated for a nerve biopsy to establish the cause of this neuropathy	
Patients that were mentally and legally capable to understand the informed consent	
Patients that signed the informed consent	

### Implantation

All patients were treated with the porcine collagen, two-component nerve guide “Neuromaix” (Matricel GmbH, Herzogenrath, Germany) that has been pre-clinically evaluated previously [12–14, 26–31]. Patients were operated under local anaesthesia, without sedation as described more detailed recently in Bozkurt et al. [25]. Briefly, patients were lying in prone position and an approximately 4–5 cm lazy-S incision was performed along the midline axis of the posterior lower leg between the lateral and medial head of the gastrocnemius muscle at the musculotendinous transition. The medial SN was separated in atraumatic fashion from the surrounding tissue by external neurolysis. Meanwhile the nerve was flushed with local anaesthetics to alleviate possible pain caused by manipulation of the nerve. For the nerve biopsy, a 20-mm nerve segment was excised and transferred to the Institute of Neuropathology on a cotton swab drenched in normal saline solution. Due to the relaxation of the nerve ends after the excision, the resulting gap size to be bridged by Neuromaix varied from 25 to 40 mm. Before implantation, Neuromaix was briefly immersed in sterile saline solution, and thereafter implanted into the nerve gap (Additional file 1: Figure S1) by using the entubulation technique as described previously (for more operational details see [25, 31]). If an additional muscle biopsy was required, 1 cm [3] of muscle tissue was harvested from the gastrocnemius or vastus lateralis muscle (*n* = 12, Table 3). All patients recovered well and responded to be free of post-operative pain within 1 week after surgery.

### Follow-up

After implantation patients were followed for up to 1 year after operation, with interim follow-up visits at 1 month, 3, 6, 9, and 12 months after operation (MPO). Safety as well as performance parameters were evaluated during every follow-up visit and compared to preoperative values and/or values measured on the same day after surgery.

Safety was evaluated by yes/no questions to the presence of typical signs of inflammation (i.e., functional loss, redness, swelling, heat, or pain) and to the presence of putrid secretion, wound dehiscence, seroma, infection, necrosis, or excessive scar tissue formation (hypertrophic scarring). If any of these questions was answered positively, the criterion “uneventful wound healing” was not accomplished.

Testing of the different sensory modalities was used to evaluate the return of sensation at the lateral aspect of the foot as listed in Table 2. To standardize the measuring of every follow-up visit, sensory testing (ST) was always performed by application of the measuring devices at the same, defined measuring point (DMP) located within the asensitive area (ASENS) [25].

**Table 2:** Sensory modalities measured by sensory testing

Sensory modality	Test	Unit
Hoffmann–Tinel’s Sign	Localization of the sign by palpation along the course of the SN	cm distance to landmarks and ASENS
Innocuous mechanoreception	Tactile: Hypoesthesia (asensitive area, ASENS)	cm^2^
Innocuous mechanoreception	Tactile: Semmes–Weinstein Monofilaments	g
Innocuous mechanoreception	Vibrotactile: vibration at 128 Hz	0–8 scale
Nociception	VAS-score, self-evaluation at the lateral foot	1–10 scale
Nociception	Presence of touch-hypersensitivity at the lateral foot	Yes/no
Spatial distribution	Pointed (sharp)/blunt discrimination	Yes/no
Thermoception	Cold/warm discrimination	Yes/no

### Hoffmann–Tinel’s Sign

Palpation of the course of the SN was used to detect positive Hoffmann–Tinel’s Sign (HTS), a spot that has been interpreted as the location of the growth cones of regenerating nerve fibers [32, 33]. The patient was asked to report, during palpation down the leg, if a spot was present located on the lower leg that elicited tingling or electrifying sensations perceived at the lateral aspect of the foot. This spot or demarcation line was marked as the HTS, and distances in relation to the lateral malleolus and middle of the metatarsus five were recorded accordingly.

### Asensitive area

The area with hypoesthesia was demarcated at every follow-up visit, by asking the patient where sensation was experienced as “normal” (i.e., compared to the non-operated foot) or diminished in response to a non-noxious touch stimulus to the ASENS. These areas were photographed and afterwards the surfaces were measured with the freely available Java-based image processing program ImageJ (RSB, imagej.nih.gov/ij). Surfaces were expressed as a percentage of the area marked on the day of surgery.

### Sensory testing

Slight touch sensation was investigated by the application of Semmes–Weinstein monofilaments (SWMT) to the DMP [34]. The patient was asked whether the filament was sensed (yes/no) after every application and slight bending of the filament as described previously (Touch Test™ Sensory Evaluators, ©Stoelting Co.). The threshold was defined as the filament that was still sensed twice during three applications. The 300 g filament was used as a cut-off value, and these values include therefore “no sensation at all” as well as “sensation at 300 g.”

Pain at the lateral foot was analyzed by self-evaluation on a numeric 1–10 VAS scale (NRS), where the score 1 reflected “no pain at all,” and 10 reflects “the maximal imaginable pain.” To verify whether allodynia or other pain-related phenomena developed over time at the lateral aspect of the foot, the patient was asked if touch of the ASENS elicited any pain sensation, and if so, to describe this sensation.

Vibrotactile sensation was determined by application of a 128 Hz tuning fork containing a 0–8 scale (Aesculap, Braun). The fork was activated and placed on the DMP, where after the patient was asked to announce when vibration was not sensed anymore. At this point, the value depicted by two intersecting triangles on the 0–8 scale was noted as previously described [35].

Spatial distribution was evaluated by responding to sharp or blunt stimuli. The patient was asked to respond to application of a 10 g (i.e., filament 5.07 in the SWMT) monofilament to the DMP. A positive blunt or sharp-sensation response was valued as an immediate response after application of the device to the DMP, when two out of three applications were correctly answered as being blunt or sharp (Neuropen^®^, Owen Mumford, Freudenberg).

Cold sensation was evaluated by asking the patient to respond to the application of a metal (cold) or a plastic (in comparison, warm) element of the Tip therm device. A positive cold-sensation response was valued as an immediate response after application of the device to the DMP, when two out of three applications were correctly classified as being cold or warm (Tip Therm^®^, Tip Therm GmbH).

### Statistical analyses

For the performance part of the study, two cases were excluded because of incomplete follow-up, and as a consequence the datasets of a total of 18 patients were used for evaluation. The non-operated side served as an intra-individual control, whereas preoperative values were used to compare the course of sensory regain in time in relation to preoperative values.

Indeed values were tested for equal variances and normality prior to statistical testing. However, due to the small *N*-number, only non-parametrical tests were performed. Comparison between operated and non-operated values was done by two-way ANOVA repeated measures with Bonferroni post hoc testing, whereas the comparison between the different time points within one side (operated vs non-operated) was performed by Kruskal–Wallis testing, with Dunn’s multiple comparison post hoc testing.

All graphs represent mean with standard error of the mean (SEM), and *p* values below 0.05 were considered as statistically significant. Statistical analysis was performed with GraphPad Prism 5 software (GraphPad Software, San Diego, CA, USA).

## Results

Twenty-three patients were screened according to the in- and exclusion criteria listed in Table 1. Three patients did not fulfill the criteria for implantation. Finally Neuromaix was implanted in a total of 20 patients after medial SN biopsy. These patients consisted of 6 female and 14 male individuals, with a mean age of 52 years ± 1.9 and ranging from 39 to 69 years. In twelve patients additional muscle biopsies (gastrocnemius, *n* = 10 and/or vastus lateralis, *n* = 2) were simultaneously performed. During the course of the study, one non-study-related severe adverse event (SAE) and three non-study-related adverse events (AE) were reported. No procedure-related, AE or SAE occurred. Two patients were lost in follow-up (Patient 003 and 010, marked with * in Table 3). Hence, for the evaluation of performance 18 datasets were included in the analysis; however, safety was evaluated for all 20 patients.

**Table 3:** Demographic data

Patient	Gender	Age	Clinical diagnosis	Muscle biopsied	Pathological diagnose
1	M	54	Sensorimotor PNP	–	Chronic neuritis, axonal neuropathy
2	F	69	Sensory PNP	–	Undefined, CIDP, or neoplasm excluded
3*	F	63	Sensorimotor PNP	GN	Chronic axonal neuropathy with neurogenic muscle atrophy
4	M	48	Sensory PNP	GN	Chronic, partially axonal, and demyelinating neuropathy with muscle atrophy
5	M	50	Sensorimotor PNP	–	Chronic, partially axonal, and demyelinating neuropathy
6	M	57	Sensory PNP	GN	Chronic, axonal neuropathy, neurogenic muscle atrophy, and perivascular inflammatory cell infiltrates in the muscle
7	M	67	Sensory PNP	VL	Chronic, predominantly axonal neuropathy with moderate demyelinating components, micro-angiopathy of endo- and epineural blood vessels, chronic neurogenic muscle atrophy, neuritis, and vasculitis-excluded, possible hereditary PNP
8	M	43	Sensory PNP	GN	Chronic, predominantly demyelinating neuropathy with axonal components, chronic neurogenic muscle atrophy, mitochondrial abnormalities
9	M	53	Sensorimotor PNP	GN	Chronic, predominantly axonal neuropathy with demyelinating components, neurogenic muscle atrophy
10*	M	46	Sensorimotor PNP	GN	Chronic, predominantly axonal neuropathy with demyelinating components, neurogenic muscle atrophy
11	M	65	Cidp	–	Chronic axonal and demyelinating neuropathy micro-angiopathy of endo- and epineural blood vessels
12	M	48	Sensory PNP	GN	Chronic axonal neuropathy with demyelinating components, micro-angiopathy of endo- and epineural blood vessels, chronic neurogenic muscle atrophy
13	M	54	Motoric PNP	GN	Chronic, axonal neuropathy, chronic neurogenic muscle atrophy
14	F	61	Sensory PNP	VL	Chronic, predominantly axonal neuropathy, with little demyelinating components, micro-angiopathy of endoneurial blood vessels, progressive neurogenic muscle atrophy
15	M	46	Sensorimotor PNP	–	Chronic, axonal neuropathy of demyelinating hypertrophic type
16	M	41	Sensorimotor PNP	GN	Chronic, axonal neuropathy, chronic neurogenic muscle atrophy
17	F	55	Sensory PNP	–	Chronic, axonal neuropathy, micro-angiopathy of endo- and epineural blood vessels, no neuritis
18	F	53	Sensorimotor PNP	–	Chronic, axonal neuropathy
19	M	39	Sensorimotor PNP	GN	Chronic, axonal neuropathy with demyelinating components, endoneurial inflammatory cell infiltrates, neurogenic muscle atrophy
20	F	47	Sensory PNP	–	Chronic, predominantly axonal neuropathy with few components, possible neuritis

**Fig. 1:**
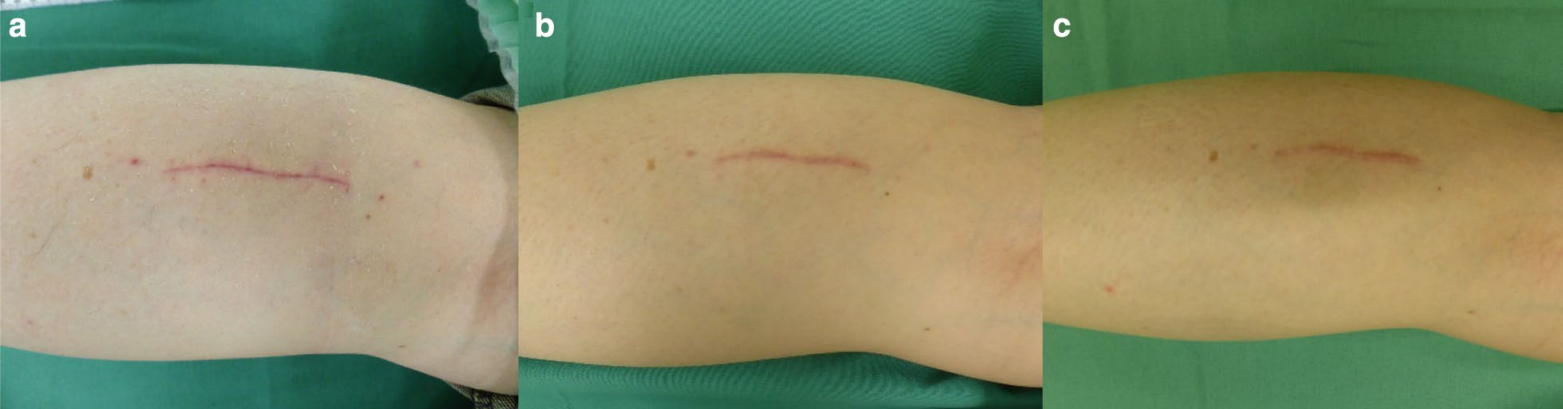
Representative example of wound healing after Neuromaix implantation. Clear wound healing was evident already after 1 month after biopsy and Neuromaix implantation (**a**). At 3 (**b**) and 6  months (**c**) and also thereafter no complications could be observed in any of the patients (Example of patient 17)

The first follow-up visit occurred at 1 month after operation (MPO). All patients investigated in this current study demonstrated clear wound healing during the complete follow-up period. Figure 1 demonstrates representative images at one, three, and 6 months after surgery. Patients did not report foreign body sensations caused by the implanted material. In addition, except for 2–3 days post-operative pain around the area of incision, they were pain-free within 1 week after surgery.

Prerequisite for including the patient’s dataset into the performance analysis was to rule out that the patient’s underlying disease may not allow formation of new sprouts. Hence, neuropathological examination of the biopsied nerve samples was used to distinguish whether newly formed axonal sprouts were present in the nerve tissue. All patients demonstrated clusters of regenerating nerve fibers in the histological preparations, indicative of an intrinsic ability of the nerve fibers to form new sprouts that may regenerate (Additional file 2: Figure S2).

**Fig. 2:**
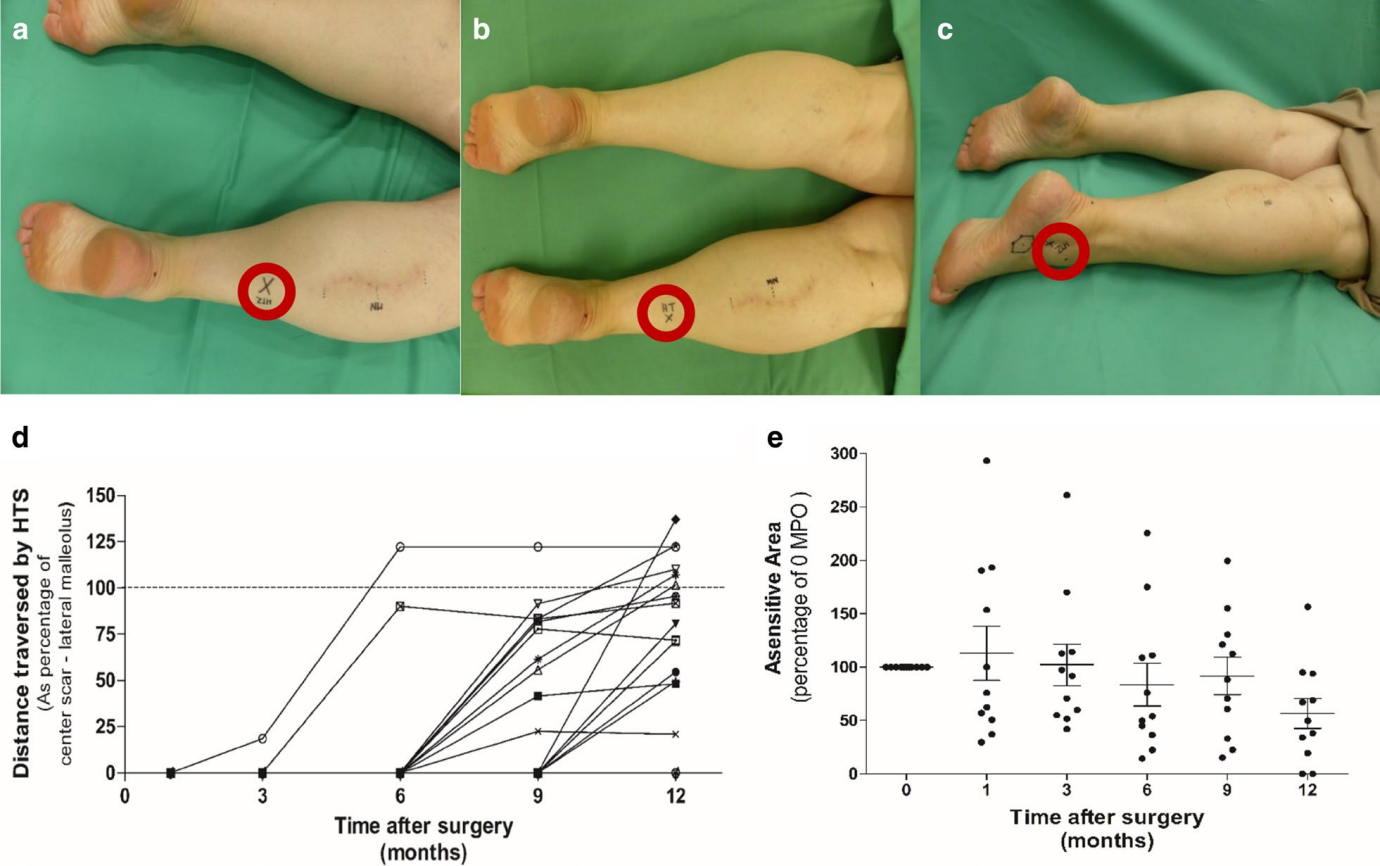
Traversing of the positive HTS in time and measurements of the ASENS. Six months after surgery a positive HTS was detected at the lower leg approximately 15 cm above the Achilles tendon (**a**). After 9 months this spot was detected more distally at approximately 10 cm distant from the Achilles tendon (**b**). Three months later this spot was detected at the level of the lateral malleolus (**c**) (Images of patient 2). Positive HTS below the operation site in the majority of patients could be observed as early as 9 months after surgery. Thereafter, the number of patients who reported positive HTS increased as well as the distance traversed by the wandering HTS. By 12 MPO, fifteen patients demonstrated a positive HTS below the operation site. Eleven of them demonstrated simultaneously a reduction in the ASENS and five patients of this latter population showed a positive HTS located below the lateral malleolus. Two patients reported no sensation of a HTS anymore, but reported complete recovery of sensation at the lateral aspect of the foot (dotted line represents HTS located at the lateral malleolus = 100%, below this line < 100% heading towards, and above this line > 100% HTS located below the lateral malleolus) (**d**). Quantification of the mean ASENS in patients with a positive HTS as percentage of the area immediately after surgery (**e**)

Representative examples of the wandering HTS in time are shown in Fig. 2a–c. Nine months after surgery, the HTS was detected as a sensitive, electrifying spot on the lower leg. Three months later this spot could be detected at the level of the lateral malleolus.

A positive HTS below the complete operation area was first detectable at 6 MPO in 2/18 patients (11%). Thereafter the percentage of patients with a positive HTS at the lower leg increased to 10/18 (56% at 9 MPO) and 15/18 (83% at 12 MPO). From these 15 patients with a positive HTS below the operation area, 11 showed simultaneously a reduction in the ASENS. Five of these 11 reported a HTS located below the lateral malleolus (28%, data points above the dotted 100% line in Fig. 2d). Immediately after surgery and at 1 MPO, the majority of patients reported complete numbness at the ASENS. Thereafter some degree of (protective) touch sensation developed over time, but often patients described a different sensation when compared to the non-operated side (i.e., most commonly described as: “delayed sensation” and/or “sensation as being covered under an asensitive layer”). ASENS did change in time in individual patients, but mean values during the 12-month follow-up did not statistically significantly differ from the status immediately after surgery (Fig. 2e). Two patients who reported a clear HTS at 9 MPO did not sense a positive HTS at 12 MPO anymore, which coincided with the recurrence of full sensation within the ASENS.

**Fig. 3:**
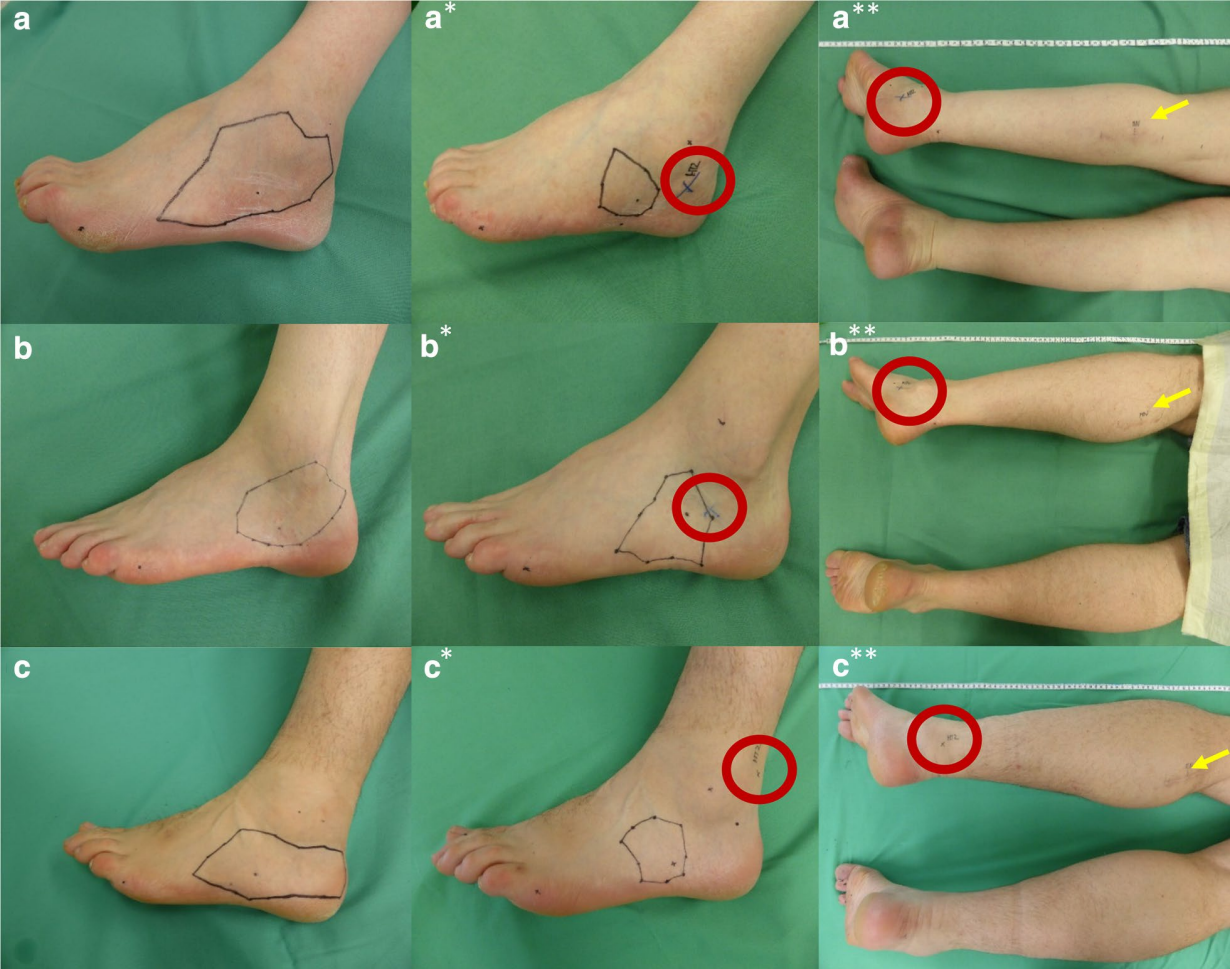
Example images of patients that showed reduction in the ASENS as well as a positive HTS that was found in the vicinity of the lateral malleolus. ASENS 1 month after surgery (**a**–**c**). Reduced ASENS at 12 MPO (a*–c*). The distance traversed by the HTS in relation to the ASENS at 12 MPO. Red circles indicate the location of the HTS, whereas the yellow arrows indicate the middle of the scar at the level of the calf (a**–c**) (Images of patient 8, 9, and 13)

Figure 3 demonstrates in a–c examples of the ASENS in relation to the location of the positive HTS 1 month after surgery, whereas a_*_–c_*_ shows a smaller ASENS at 12 MPO. In addition, Fig. 3a**, b**, c** visualizes the distance traversed by the HTS in relation to the ASENS at 12 MPO (Red circles show the location of the HTS and yellow arrows the location of the middle of the scar, respectively).

**Fig. 4:**
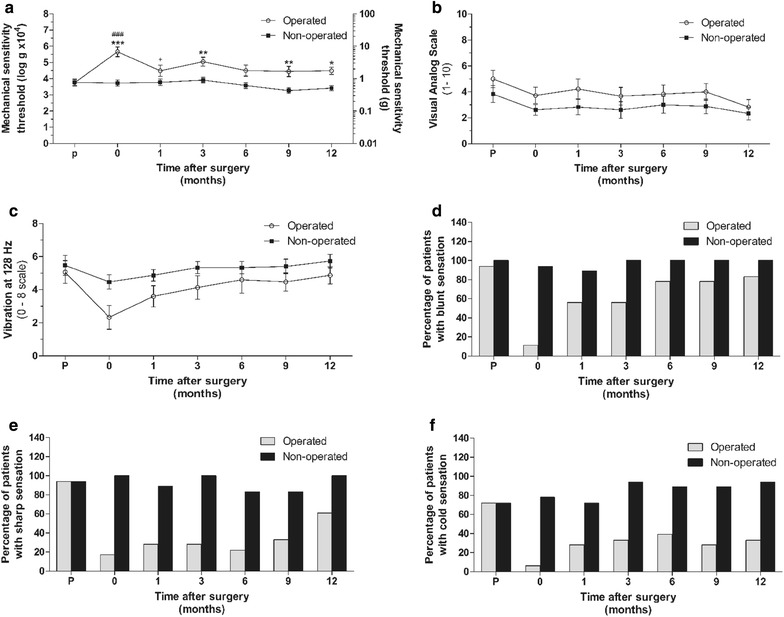
Mechanical sensation was reduced immediately after the operation, and patients often reported “no sensation at all.” Thereafter mechanical sensitivity thresholds slightly improved, but never reached values measured at the non-operated side (**a**). VAS scores remained constant over time on both non-operated and operated feet (**b**). Vibrotactile sensation was slightly reduced after surgery (ns). In time, vibrotactile sensation recovered, but remained reduced compared to the non-operated foot (**c**). Blunt sensation returned quite rapid after surgery; by 1 MPO slight touch with the 10 g filament elicited in more than 50% of the patients a positive response (**d**). Sharp sensation recovered slower; only in the last month there was a clear improvement in the majority of patients detectable (**e**). Cold sensation showed a slower recovery in time, and even after 12 MPO the majority of patients were unable to sense the cold metal tip (**f**). Data of **a**–**c** represent mean ± SEM where */**/*** represent *p* < 0.05/0.01/0.001 operated vs non-operated, ^###^ represents *p* < 0.001 preoperative vs post-surgery, + represents *p* < 0.05 post-surgery compared to immediately after surgery (0 MPO). Graphs **d**–**f** represent percentages of total

Prior to operation, mechanical sensation at both feet was similar (operated, open circles: 3.8 ± 0.3, non-operated, filled squares: 3.8 ± 0.2, Fig. 4a). After the operation, a clear elevation of the mechanical sensitivity threshold could be observed at the operated foot when compared to the non-operated side (operated: 5.8 ± 0.4, non-operated: 3.8 ± 0.2, ***p < 0.001). Immediately after surgery, thresholds were in the range of “loss of protective sensation.” At this time point, patients often responded with “no sensation at all” (53%, deep pressure sensation only). This post-operative elevation of the mechanical sensitivity threshold faded in time, but thresholds remained higher than compared to the non-operated side (Fig. 4a, **p < 0.01 at 3 and 9 MPO and at 12 MPO; operated: 4.5 ± 0.3 open circles, non-operated 3.5 ± 0.2, filled squares *p < 0.05). This coincided with patients reporting return of protective sensation (i.e., complete numbness changed into some degree of touch sensation).

Patients displayed post-operative pain around the operation site during the first 2 days after surgery, but after 1 week, patients were completely free of any pain. Over time patients did not show strong fluctuations in pain sensation at the lateral foot (Fig. 4b). VAS scores prior to surgery were slightly elevated for the operated side (5.2 ± 0.7, open circles) when compared to the non-operated side (4.0 ± 0.8, *p > 0.05, filled squares). None of the patients reported pain, but rather reported numbness, or tingling sensations after touch of the ASENS. Immediately after surgery, a drop in vibration sensation (Fig. 4c) could be observed at the operated side compared to the preoperative status, but this reduction was not statistically significant (preoperative: 5.1 ± 0.8 and 0 MPO: 2.4 ± 0.9). Vibration sensation returned (12 MPO: operated: 5.0 ± 0.6, open circles), but remained slightly lower than at the non-operated side (12 MPO: 5.7 ± 0.4, filled squares).

Figure 4d demonstrates the percentage of patients that perceived blunt stimuli (10 gr SWT filament) in time. Blunt sensation returned quite rapidly after surgery, as with 1 MPO approximately halve of the patients (56%) sensed the 10 g SWT filament. After 12 MPO, this percentage was increased to 83%. Sharp sensation (pin-prick) did not recover as quickly as blunt sensation after surgery; up to 9 MPO less than half of the patients were able to detect the sharp stimulus (33%). During the last 3 months, a clear recovery could be observed, as finally at 12 MPO 61% of the patients were able to detect sharp stimuli (Fig. 4e). The number of patients who detected the cold stimulus increased only little in time. Even after 12 MPO the majority of patients were unable to detect cold stimuli (33% Fig. 4f).

## Discussion

To date there is still an urgent clinical request for alternatives to the autologous nerve transplantation for the repair of damaged peripheral nerves [1–3]. These alternatives can exist of biomaterials having the advantage that they can be used off the shelf. In this present study we evaluated the newly developed collagen-based nerve guide Neuromaix in a first in human study. As a two-component scaffold, Neuromaix was implanted into 20–40 mm sural nerve biopsy defects to support functional axonal regeneration across this nerve gap [25]. Safety as well as performance parameters of this application of the scaffold were evaluated up to 1 year after the implantation.

Our data first of all show that Neuromaix is safe; uneventful wound healing was evident in every patient during the complete follow-up of this study. Our findings are in line with previous reports about collagen materials being well tolerated by the human body, in general [36] and more specifically after implantation as a tube in human PNI [37]. Moreover, our own findings for Neuromaix in animal experiments of PNI complement these current *first in human* results [12–14, 26–31]. In addition, patients did not complain about foreign body sensations caused by the material. Except for a few days post-operative pain (maximally 1 week), patients did not develop any persistent pain around the scar area or at the lateral foot. Most commonly sensation after touch at the ASENS was described as “delayed” or “covered by an asensitive layer” hypoesthesia, but never as painful to a non-painful stimulus (mechanical allodynia), or as burning or stinging pain (hyperalgesia).

Second, our data support the notion that implantation of Neuromaix may serve as a standard repair method to overcome secondary complications after nerve biopsy [37–41]. Biopsy removal at the level of the calf, in comparison to the classical method at the lateral malleolus [39], provides, as our data confirm, ideal wound healing conditions with excellent soft tissue covering. This method provides the opportunity to obtain muscle biopsies at the same time, without losing diagnostic value of the biopsies taken [42, 43] and providing the most optimal wound healing conditions for the patient, as already discussed previously [25]. In addition, the majority of the patients reported spontaneous tingling and electrifying sensations, which has been associated with movement of the growth cone, but no pain between follow-up visits [32, 33].

The nerve’s intrinsic ability to regenerate is, of course, a prerequisite for the evaluation of performance. Patients assigned for a nerve biopsy concern a population that has already been examined clinically by the standard diagnostic work-up for PNP, showing no abnormalities in blood/spinal fluid counts, though often demonstrating abnormal electrophysiological parameters (i.e., impaired amplitudes and motor/sensory nerve conductance velocities). By semi-thin resin section histology of the biopsy material, we confirmed the presence of regeneration clusters in every patient, indicating the intrinsic capability of the nerve to regenerate. Thus, all patients with complete follow-up could be included into the performance analysis (*n* = 18). However, we noticed by tracking the HTS in time that regeneration rates in our patients were slower than the 1 mm/day frequently reported in literature for healthy persons [44]. Our data confirm that the patient’s underlying disease condition is an important factor influencing nerve regeneration [45]. Therefore the patient’s underlying condition should be taken into account all time while planning and evaluating data in this nerve injury model, as it has an important influence on regeneration and the timing of the return of sensation.

Moreover another important factor in our study to consider is the age of our patient population (mean age 52 years ± 1.9, range 39–69 years). Previous work from others already showed that age is an important factor influencing myelin- and axonal-related parameters, the proportions of different fiber types in the nerve, and electrophysiological properties (Summarized in Verdu 2000 [45]). It can be expected that variability in age may also account for the individual differences observed in our patients with regard to the timing and degree of returning of sensation.

Indeed biopsy and implantation at the level of the calf is located more distant from the target organ than at the ankle [37–41], and therefore regenerating nerve fibers have to traverse a longer distance to re-innervate the lateral skin of the foot. It can be expected that this has also implications for the timing of return of sensation. However, we were able to detect positive HTS in fifteen out of eighteen patients by 12 MPO. Moreover eleven of them demonstrated a HTS below the operation area with simultaneous reduction in the ASENS, suggesting that nerve fibers once regenerated across Neuromaix, entered the former trajectories of the SN [32, 33]. Five of the latter demonstrated positive HTS that passed the lateral malleolus heading towards the lateral aspect of the foot and two of them reported complete return of sensation by 12 MPO.

All patients reported reduced sensation in the area innervated by the SN immediately after operation. This was confirmed by the testing of various sensory modalities. Some degree of protective touch sensation returned in all patients. Sharp and cold sensation recovered more slowly than touch and vibration sensation. As previously mentioned, timing of the return of sensation has been shown to depend on age, gender, underlying disease of the patient, time to repair and the type, location, and extend of injury [46]. This may partly account for the individual differences in recovery seen in our population (i.e., here influenced by age, gender, and underlying disease). With respect to the different sensory modalities, thresholds generally increase during aging [45]. Comparison to other repair approaches is difficult, as the timing of, and the extent of sensory regain highly depends on the size of the nerve gap to be bridged (i.e., < 15 mm or overcritical), the distance to be traversed by the regenerating axons (i.e., distant or near the target skin areal) as well as the location of the area to be re-innervated (i.e., hand palms/foot soles, or dorsolateral foot). For the lower extremities, the greatest alterations in sensation after PNI have been reported to manifest as loss of mechanical pressure, vibration, and cold detection [47]. Hyperesthesia as well as hyperalgesia were less frequently seen. In addition pain-related symptoms were detected with reduced thresholds to cold and pressure pain. In contrast, warm pain sensation showed elevated thresholds [47]. Loss of touch, vibration, and cold sensation was also evident in our patient population after surgery, but steadily improved over time.

Eleven of fifteen patients with a positive HTS at the lower leg demonstrated simultaneously a reduction in the ASENS, suggesting that newly regenerated fibers re-innervated the skin areal of the lateral foot. As these observations were not concurred by pain-related symptoms at the ASENS, implantation of Neuromaix could become a standard procedure after nerve biopsy to overcome possible co-morbidities. Restoration the transmission of sensory information from the periphery to the brain has been suggested to alleviate pain-related symptoms, such as allodynia [48, 49]. This method can even be extrapolated to conditions where harvesting of donor nerve material for transplantation leaves a nerve gap.

Electrophysiological recordings and neuro-imaging techniques however are needed to complement these first results on sensory regain [50, 51]. Nevertheless, our data provide promising prospective for the reconstruction of combined nerves, which should be tested in larger, randomized, multi-center, clinical trials in the near future.

## Additional files

**Additional file 1.** Example of a high-resolution ultrasound image of Neuromaix one month after implantation in the SN biopsy gap. Neuromaix, existing of Epimaix and Perimaix, was clearly detectable between the proximal and distal nerve stumps one month after implantation in the SN biopsy gap (Example of patient 001; Scale bar: 5 mm). At the right SEM images of the Neuromaix nerve guide.

**Additional file 2.** Examples of toluidine blue stained SN biopsies of the current patient population. Histological examination of the nerve biopsies demonstrated that every patient exhibited regeneration clusters, small groups of densely clustered fibers with thin myelination (black arrows in A–D and encircled group of fibers in the magnification, white arrows indicate small diameter sensory fibers, scale bar 20 µm). This effect that is frequently observed as a compensatory mechanism in neuropathy, points out the intrinsic ability of the axons to sprout and potentially regenerate (Semi-thin sections, toluidine blue; scale bar = 20 µm examples of patients 002, 008, 009, and 005 respectively).

## Abbreviations

ANT: autologous nerve transplantation
ASENS: asensitive area
DMP: defined measuring point
GN: gastrocnemius muscle
HTS: Hoffmann–Tinel’s Sign
MPO: months after operation
NRS: numeric rating scale
PNI: peripheral nerve injury
PNP: polyneuropathy
ST: sensory testing
SN: sural nerve
SWMT: Semmes–Weinstein monofilament test
VAS: visual analog scale
VL: vastus lateralis
