# Supplementary figures and images for: Clinical and biometrical 12-month follow-up in patients after reconstruction of the sural nerve biopsy defect by the collagen-based nerve guide Neuromaix
Source: Eur J Med Res. 2017 Sep 22;22:34. doi: 10.1186/s40001-017-0279-4 (PMC5610476; doi:10.1186/s40001-017-0279-4)

## Slide 1
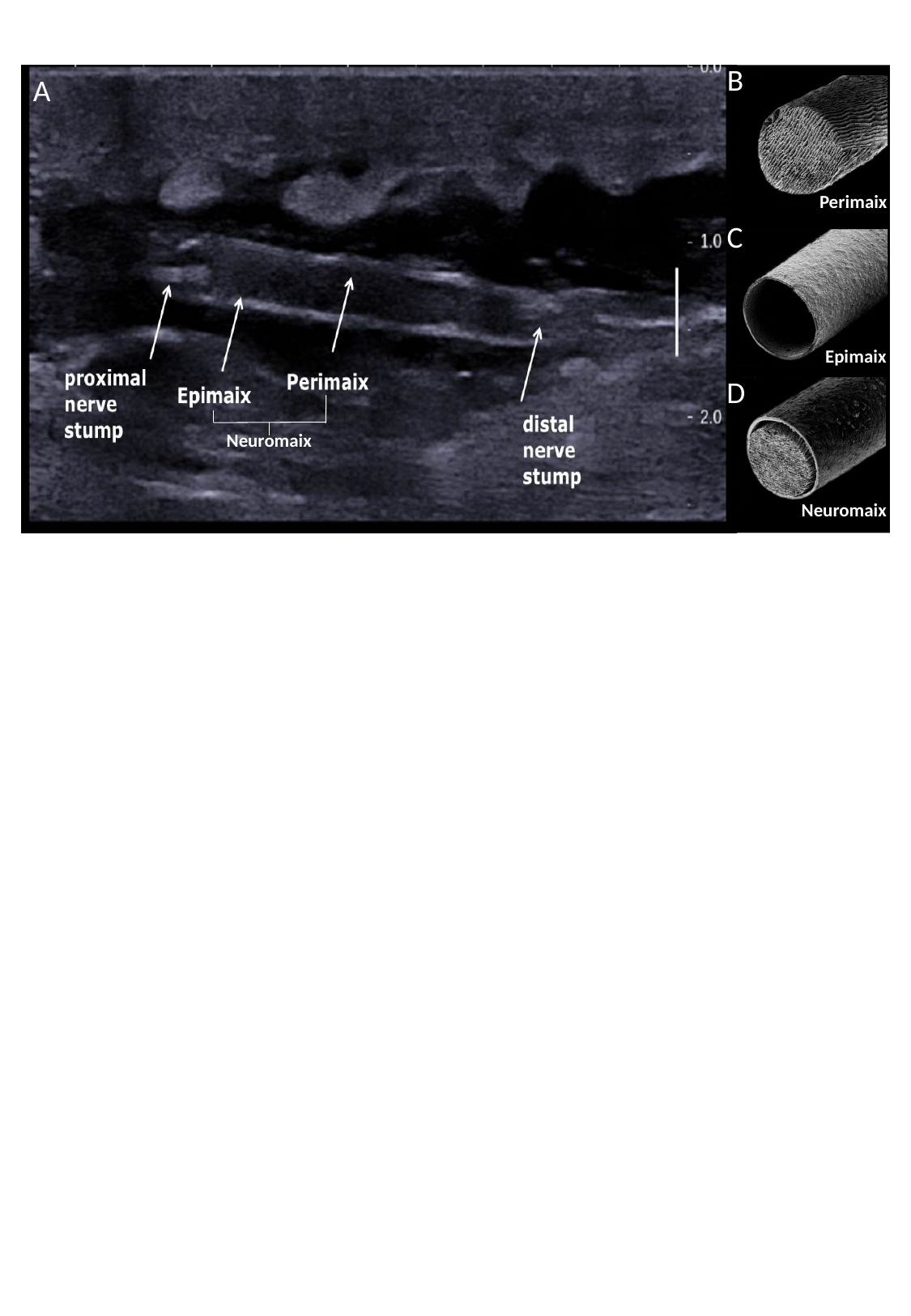

B
A
Perimaix
C
Epimaix
D
Neuromaix
Neuromaix

Supplement: Supplementary file 1 — Additional file 1. Example of a high-resolution ultrasound image of Neuromaix one month after implantation in the SN biopsy gap. Neuromaix, existing of Epimaix and Perimaix, was clearly detectable between the proximal and distal nerve stumps one month after implantation in the SN biopsy gap (Example of patient 001; Scale bar: 5 mm). At the right SEM images of the Neuromaix nerve guide. [file 40001_2017_279_MOESM1_ESM.pptx]

## Slide 1
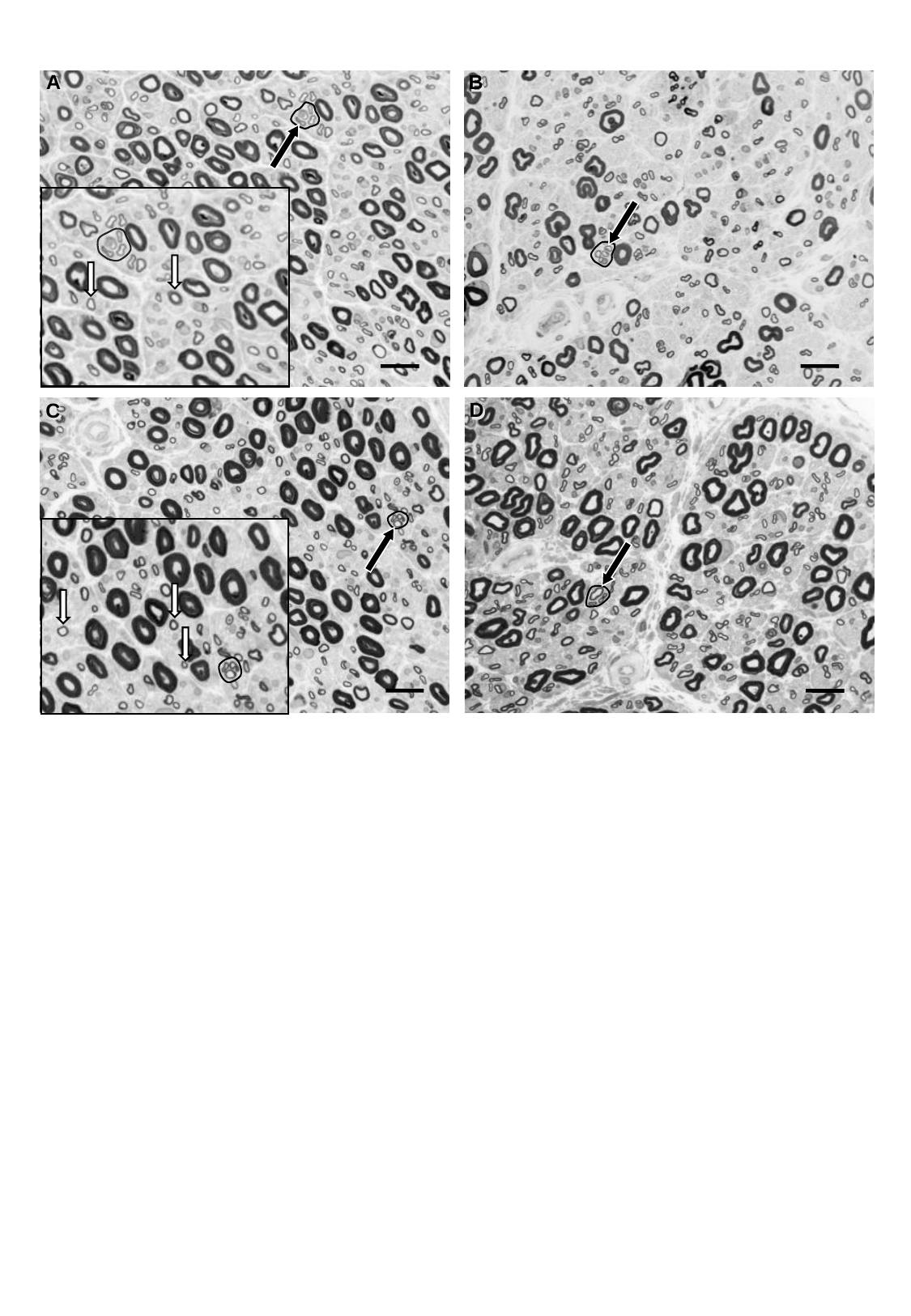

A
B
D
C

Supplement: Supplementary file 2 — Additional file 2. Examples of toluidine blue stained SN biopsies of the current patient population. Histological examination of the nerve biopsies demonstrated that every patient exhibited regeneration clusters, small groups of densely clustered fibers with thin myelination (black arrows in A–D and encircled group of fibers in the magnification, white arrows indicate small diameter sensory fibers, scale bar 20 µm). This effect that is frequently observed as a compensatory mechanism in neuropathy, points out the intrinsic ability of the axons to sprout and potentially regenerate (Semi-thin sections, toluidine blue; scale bar = 20 µm examples of patients 002, 008, 009, and 005 respectively). [file 40001_2017_279_MOESM2_ESM.ppt]
